# Prediction Model for Freedom from TLR from a Multi-study Analysis of Long-Term Results with the Zilver PTX Drug-Eluting Peripheral Stent

**DOI:** 10.1007/s00270-020-02648-6

**Published:** 2020-10-06

**Authors:** Michael D. Dake, Fabrizio Fanelli, Aaron E. Lottes, Erin E. O’Leary, Heidi Reichert, Xiaohui Jiang, Weiguo Fu, Osamu Iida, Kan Zen, Marc Schermerhorn, Thomas Zeller, Gary M. Ansel

**Affiliations:** 1 The University of Arizona, Health Sciences Innovation Building, 1670 East Drachman Street, 9th Floor SVP Suite, P.O. Box 210216, Tucson, AZ 85721-0216 USA; 2 Department of Vascular and Interventional Radiology, “Careggi” University Hospital, Florence, Italy; 3 Purdue University, West Lafayette, IN USA; 4 Cook Research Incorporated, West Lafayette, IN USA; 5 EpidStrategies, Ann Arbor, MI USA; 6 Department of Vascular Surgery, Zhongshan Hospital, Fudan University, Shanghai, China; 7 Cardiovascular Center, Kansai Rosai Hospital, Amagasaki, Japan; 8 Department of Cardiovascular Medicine, Graduate School of Medical Science, Kyoto Prefectural University of Medicine, Kyoto, Japan; 9 Division of Vascular Surgery, Department of Surgery, Beth Israel Deaconess Medical Center, Boston, MA USA; 10 Universitaets-Herz-Zentrum Freiburg – Bad Krozingen, Bad Krozingen, Germany; 11 Department of Medicine, Ohio Health/Riverside Methodist Hospital, Columbus, OH USA

**Keywords:** Drug-eluting stent, Paclitaxel, Peripheral artery disease, Prediction model, Target lesion revascularization

## Abstract

**Purpose:**

Develop a prediction model to determine the impact of patient and lesion factors on freedom from target lesion revascularization (ffTLR) for patients who are candidates for Zilver PTX drug-eluting stent (DES) treatment for femoropopliteal lesions.

**Methods:**

Patient factors, lesion characteristics, and TLR results from five global studies were utilized for model development. Factors potentially associated with TLR (sex, age, diabetes, hypertension, hypercholesterolemia, renal disease, smoking status, Rutherford classification, lesion length, reference vessel diameter (RVD), popliteal involvement, total occlusion, calcification severity, prior interventions, and number of runoff vessels) were analyzed in a Cox proportional hazards model. Probability of ffTLR was generated for three example patient profiles via combinations of patient and lesion factors. TLR was defined as reintervention performed for ≥ 50% diameter stenosis after recurrent clinical symptoms.

**Results:**

The model used records from 2227 patients. The median follow-up time was 23.9 months (range: 0.03–60.8). The Kaplan–Meier estimates for ffTLR were 90.5% through 1 year and 75.2% through 5 years. In a multivariate analysis, sex, age, Rutherford classification, lesion length, RVD, total occlusion, and prior interventions were significant factors. The example patient profiles have predicted 1-year ffTLRs of 97.4, 92.3, and 86.0% and 5-year predicted ffTLRs of 92.8, 79.5, and 64.8%. The prediction model is available as an interactive web-based tool (https://cooksfa.z13.web.core.windows.net).

**Conclusions:**

This is the first prediction model that uses an extensive dataset to determine the impact of patient and lesion factors on ffTLR through 5 years and provides an interactive web-based tool for expected patient outcomes with the Zilver PTX DES.

**Clinical Trial Registrations:**

Zilver PTX RCT unique identifier: NCT00120406; Zilver PTX single-arm study unique identifier: NCT01094678; Zilver PTX China study unique identifier: NCT02171962; Zilver PTX US post-approval study unique identifier: NCT01901289; Zilver PTX Japan post-market surveillance study unique identifier: NCT02254837.

**Levels of Evidence:**

Zilver PTX RCT: Level 2, randomized controlled trial; Single-arm study: Level 4, large case series; China study: Level 4, case series; US post-approval study: Level 4, case series Japan PMS study: Level 4, large case series.

**Electronic supplementary material:**

The online version of this article (10.1007/s00270-020-02648-6) contains supplementary material, which is available to authorized users.

## Introduction

The management of symptomatic peripheral artery disease (PAD) is frequently a complex challenge influenced by a variety of patient factors and anatomic characteristics of the disease. In an effort to reduce restenosis, the most common cause of failure, following endovascular intervention, drug-eluting stents (DES) were developed [1, 2] with the hope of providing a safe and durable endovascular option for treatment of patients with PAD.

Endovascular drug-based therapies for PAD have consistently shown superior patency and freedom from target lesion revascularization (ffTLR) outcomes relative to traditional devices [e.g., standard percutaneous balloon angioplasty (PTA) and bare metal stents (BMS)] [3, 4]. These drug-based therapies have demonstrated long-term effectiveness [5, 6].

Despite improved results with drug-based technologies, a limitation of these endovascular devices is still restenosis. Patient-level data of coronary DES have been pooled to analyze which factors are predictors for revascularization [7]. In terms of risk factors for revascularization after femoropopliteal DES therapy, including patient demographic and clinical variables, limited data exist. The purpose of this study is to develop a prediction model using patient-level data from five prospective clinical trials to determine the impact of patient and lesion factors on ffTLR for patients who are candidates for DES treatment.

## Methods

The global clinical program for the DES (Zilver PTX, Cook Medical, Bloomington, IN, USA) consists of multiple Cook-sponsored pre-market (i.e., Zilver PTX randomized controlled trial [RCT], single-arm study [SAS], and China study) and post-market clinical (i.e., US post-approval study [US PAS] and Japan post-market surveillance study [PMS]) studies. A detailed description of the study design, inclusion and exclusion criteria, and results have been previously published for three of the five trials included in this analysis [1, 5, 8–11]. Table 1 describes the study characteristics for the five studies included in the current analysis.

**Table 1:** Global clinical studies for the Zilver PTX DES

	RCT	SAS	China	US PAS	Japan PMS
Study design	Prospective, multicenter, randomized controlled trial	Prospective, multicenter, single-arm study	Prospective, multicenter, single-arm study	Prospective, multicenter, single-arm study	Prospective, multicenter, single-arm study
Number of DES patients	305	787	178	200	904
Enrollment dates	March 2005 through August 2008	April 2006 through June 2008	July 2014 through December 2015	August 2013 through November 2015	May 2012 through February 2013
Follow-up	5 years (complete)	2 years (complete)	1 years (complete)	5 years (ongoing)	5 years (complete)
Geography	United States, Japan, Germany	Europe, Korea, Canada	China	United States	Japan
Pre-market versus post-market	Pre	Pre	Pre	Post	Post
*Key study criteria*					
Inflow tract stenosis	No significant untreated inflow tract stenosis	No significant untreated inflow tract stenosis	No significant untreated inflow tract stenosis	No significant untreated inflow tract stenosis	*
Number of patent runoff vessels	≥ 1	≥ 1	≥ 1	≥ 1	*
Prior stent treatment in SFA	No	Allowed	No	No	*
Lesion length	≤ 140 mm	No exclusion (maximum of 4 DES per patient)	≤ 140 mm	≤ 140 mm per limb and280 mm per patient	*
Number of lesions	One lesion per limb	No exclusion (maximum of 4 DES per patient)	One lesion per patient	Two lesions per patient	*
Renal disease	Excluded if serum creatinine > 2.0, renal failure, or dialysis	No exclusions	Excluded if in chronic renal failure (eGFR < 30 mLs/ min/1.73 m^2^), or is on hemodialysis or chronic peritoneal dialysis	Chronic renal failure was not excluded	*
Core laboratory	Angiography Duplex ultrasound X-ray	X-ray^a^	Angiography Duplex ultrasound	Angiography Duplex ultrasound X-ray	X-ray^a^
CEC	Yes	Yes	No	Yes	Yes
DSMB	Yes	Yes	No	No	No

*CEC* clinical events committee; *DSMB* data safety monitoring board; *SFA* superficial femoral artery

*All consecutive patients treated with the DES were enrolled (up to enrollment limit); no exclusion criteria

^a^In the event a stent fracture was reported by an investigative site, an independent core laboratory reviewed the imaging, confirmed the fracture, and classified the fracture by type (I–IV)

Data from the five global studies of the DES were combined for the post hoc analysis based on factors that were defined consistently across and the availability of these factors to address differential patient risk for TLR across all studies. Common patient-level characteristics hypothesized to be associated with TLR and utilized for model development included sex, age (< 65, 65–74, 75–84, ≥ 85 years), diabetes, hypertension, hypercholesterolemia, renal disease (e.g., hematuria, chronic urinary tract infections, renal calculi, renal insufficiency), smoking history (never, past, current), Rutherford classification (claudicant, critical limb ischemia [CLI]), lesion length (50 mm buckets, from < 50 mm to ≥ 300 mm), reference vessel diameter (< 5 mm vs. ≥ 5 mm), popliteal involvement, total occlusion, calcification severity (none, mild/moderate, severe), and prior intervention of the study lesion. When available, core laboratory data were used. The continuous patient-level characteristics (i.e., age, Rutherford classification, lesion length, and reference vessel diameter) were categorized in order to facilitate the prediction model and limit the number of combinations. TLR was defined in all studies as reintervention performed for ≥ 50% diameter stenosis after recurrent clinical symptoms. As pre-specified, 5-year follow-up was defined as the window of 4.5 years through the end of the follow-up window.

Baseline characteristics were summarized using frequencies and percentages. A Kaplan–Meier analysis was performed to assess overall freedom from TLR. Cox proportional hazards models were fit to predict freedom from TLR using both a univariate and multivariate approach. Validation was performed using a 60/40 training/test split of the dataset to evaluate model discrimination and calibration [12]. A 60/40 split was chosen in order to provide sufficient observations in each set, while preventing possible overfitting of the training model. The splits were created by drawing a random sample of observations stratified by study. Receiver operating curves (ROCs) for the complete data set and for training data set at 1, 3, and 5 years were generated. A concordance measure derived from the 40% test set was used. Model calibration was gauged using predicted survival plots for the training set versus the test set based on three calibration groups defined by cuts at the 27th and 73rd percentiles of the distribution of the linear predictor [13].

Probability of freedom from TLR with 95% confidence intervals (CI) was generated from the validated model at 1, 2, 3, 4 and 5 years for combinations of patient and lesion factors composing a patient profile. This prediction model is available as an interactive web-based tool (https://cooksfa.z13.web.core.windows.net). Analyses were conducted using SAS software, Version 9.4 of the SAS System for Windows, and Stata version 16.

Authors had access to the patient-level data used in the current analysis.

## Results

**Fig. 1:**
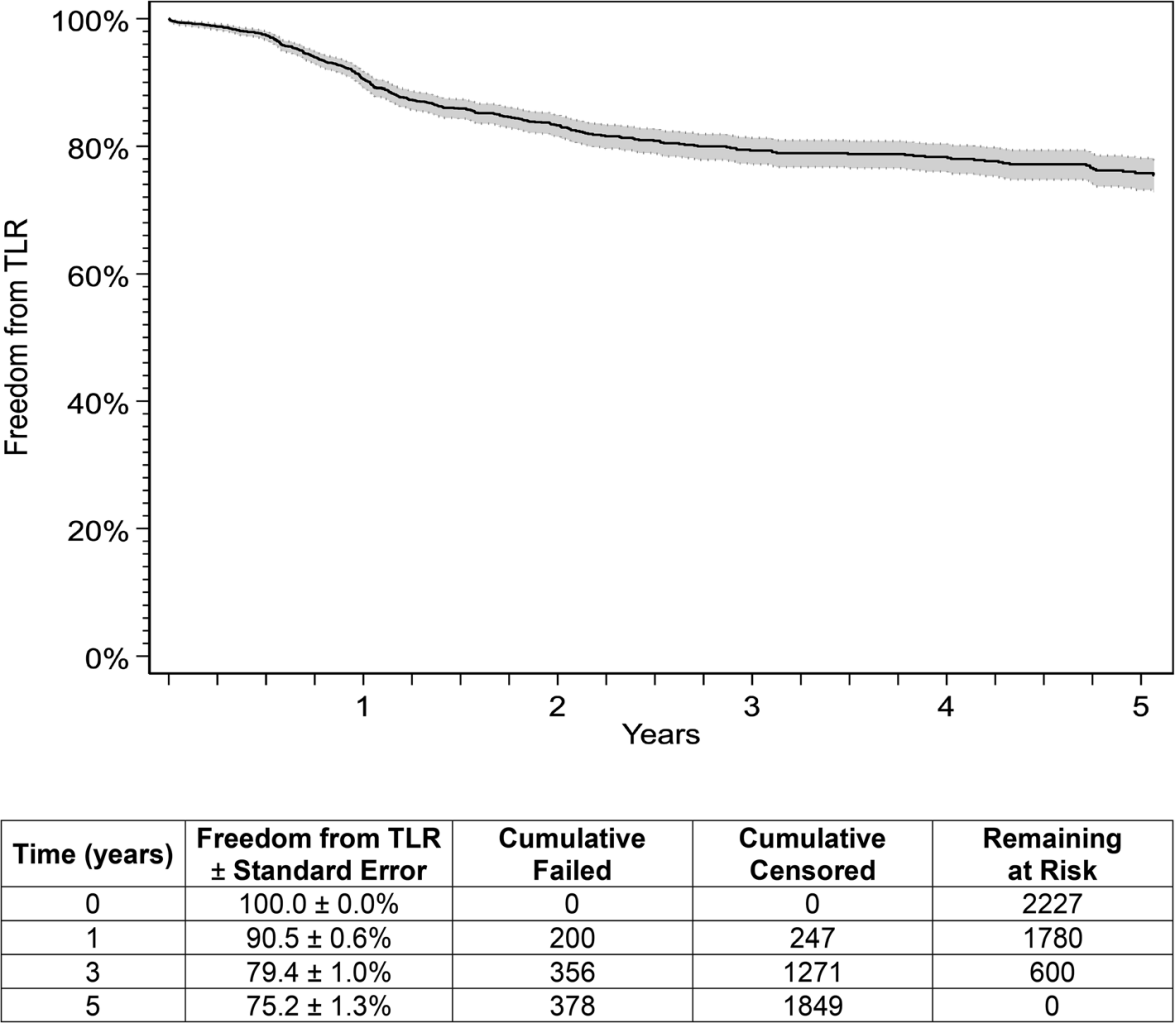
Kaplan–Meier curve for freedom from TLR. Five-year freedom from TLR outcomes and 95% confidence intervals for the DES across five clinical studies. The life table is included. DES, drug-eluting stent TLR, target lesion revascularization

Overall, there were 2227 out of 2374 (94%) cases with complete data used for the analysis. The median follow-up time was 23.9 months (range: 0.03–60.8 months), with 61,489 months of total time at risk. For this cohort of patients, there were 1780 (79.9%), 600 (26.9%) and 443 (19.9%) patients with follow-up through 1, 3 and 5 years, respectively. The Kaplan–Meier estimates for freedom from TLR were 90.5% through 1 year and 75.2% through 5 years (Fig. 1). Table 2 shows baseline patient demographics and lesion characteristics both by individual study and overall. Hypercholesterolemia in China was notably low (18.9%) compared to the rates reported in other studies. The rates of smoking status were variable by study. Due to study design, lesions were longer in the single-arm study as well as in the Japan post-market study. In addition, treatment of in-stent restenosis (ISR), which accounts for 60% of prior interventions, was permitted in the single-arm study as well as in the Japan post-market study. A comparison of the distribution of predictors between cases included in the model versus those omitted is shown in Supplementary Table 1. The average RVD in the cohort of patients omitted was larger than the average RVD retained in the model; this distribution of vessel sizes was significantly different (*p* = 0.001).

**Table 2:** Baseline patient demographics and lesion characteristics

Characteristic^a^	RCT (*n* = 301)	SAS (*n* = 707)	China (*n* = 175)	US PAS (*n* = 200)	Japan PMS (*n* = 844)	Overall (*n* = 2227)
Sex						
Male	67.1% (202)	74.4% (526)	78.9% (138)	63.0% (126)	70.0% (591)	71.1% (1583)
Female	32.9% (99)	25.6% (181)	21.1% (37)	37.0% (74)	30.0% (253)	28.9% (644)
Age						
< 65	39.2% (118)	38.6% (273)	40.6% (71)	37.0% (74)	15.6% (132)	30.0% (668)
65–74	33.9% (102)	40.3% (285)	34.9% (61)	38.5% (77)	37.2% (314)	37.7% (839)
75–84	24.6% (74)	20.1% (142)	21.1% (37)	21.0% (42)	39.3% (332)	28.2% (627)
> 85	2.3% (7)	1.0% (7)	3.4% (6)	3.5% (7)	7.8% (66)	4.2% (93)
Diabetes	48.2% (145)	35.9% (254)	55.4% (97)	46.0% (92)	59.5% (502)	48.9% (1090)
Hypertension	87.7% (264)	79.3% (561)	76.6% (134)	93.5% (187)	85.4% (721)	83.8% (1867)
Hypercholesterolemia	76.1% (229)	58.1% (411)	18.9% (33)	86.5% (173)	61.1% (516)	61.2% (1362)
Renal disease^b^	9.0% (27)	11.3% (80)	5.7% (10)	13.0% (26)	43.2% (365)	22.8% (508)
Smoking status						
Never	13.6% (41)	17.1% (121)	42.3% (74)	15.5% (31)	36.4% (307)	25.8% (574)
Past	56.1% (169)	49.4% (349)	25.1% (44)	43.0% (86)	45.3% (382)	46.3% (1030)
Current	30.2% (91)	33.5% (237)	32.6% (57)	41.5% (83)	18.4% (155)	28.0% (623)
Rutherford						
Claudicant	92.4% (278)	89.5% (633)	92.0% (161)	86.0% (172)	78.0% (658)	85.4% (1902)
CLI	7.6% (23)	10.5% (74)	8.0% (14)	14.0% (28)	22.0% (186)	14.6% (325)
Lesion length						
< 50 mm	46.2% (139)	27.6% (195)	35.4% (62)	27.5% (55)	14.6% (123)	25.8% (574)
50–99 mm	33.9% (102)	28.0% (198)	36.0% (63)	49.5% (99)	17.2% (145)	27.3% (607)
100–149 mm	17.9% (54)	15.8% (112)	18.3% (32)	17.0% (34)	19.8% (167)	17.9% (399)
150–199 mm	1.7% (5)	8.6% (61)	9.1% (16)	4.0% (8)	9.4% (79)	7.6% (169)
200–249 mm	0.3% (1)	8.9% (63)	1.1% (2)	1.0% (2)	18.0% (152)	9.9% (220)
250–299 mm	0.0% (0)	9.2% (65)	0.0% (0)	1.0% (2)	9.7% (82)	6.7% (149)
> 300 mm	0.0% (0)	1.8% (13)	0.0% (0)	0.0% (0)	11.4% (96)	4.9% (109)
RVD						
< 5 mm	42.2% (127)	19.7% (139)	69.1% (121)	40.5% (81)	19.2% (162)	28.3% (630)
≥ 5 mm	57.8% (174)	80.3% (568)	30.9% (54)	59.5% (119)	80.8% (682)	71.7% (1597)
Popliteal involvement	5.6% (17)	9.8% (69)	2.3% (4)	8.0% (16)	7.9% (67)	7.8% (173)
Total occlusion	32.6% (98)	42.9% (303)	50.3% (88)	36.5% (73)	44.4% (375)	42.1% (937)
Calcification						
None	24.9% (75)	19.4% (137)	24.6% (43)	13.5% (27)	28.1% (237)	23.3% (519)
Mild/moderate	60.5% (182)	60.0% (424)	65.7% (115)	69.5% (139)	53.8% (454)	59.0% (1314)
Severe	14.6% (44)	20.7% (146)	9.7% (17)	17.0% (34)	18.1% (153)	17.7% (394)
Prior interventions	5.3% (16)	25.2% (178)	1.1% (2)	12.5% (25)	29.1% (246)	21.0% (467)
Number of runoff vessels						
0–1	22.9% (69)	18.1% (128)	38.3% (67)	22.5% (45)	39.0% (329)	28.6% (638)
> 2	77.1% (232)	81.9% (579)	61.7% (108)	77.5% (155)	61.0% (515)	71.4% (1589)

*CLI* critical limb ischemia; *RVD* reference vessel diameter

^a^Best available data was used since a core lab was not utilized in all studies

^b^The status for renal disease was collected as yes/no for all studies except the China study where it was collected as “chronic renal failure” (*n* = 0), “dialysis” (*n* = 0) or “other renal disease” (*n* = 10). The sum of these three measured were considered for renal disease status for the China study

Results from the univariate Cox models for factors related to reinterventions are shown in Table 3. In these analyses, diabetes, hypertension, hypercholesterolemia, RVD, calcification severity, and number of runoff vessels were not significant. To account for differences in the distribution of some patient risk factors across studies, all characteristics were included in the multivariate Cox model (Table 4). In the multivariate analysis, sex, age, Rutherford classification, lesion length, RVD, total occlusion, and prior interventions were significant factors. The assumption of proportionality for the multivariate Cox model was met (global test, *p* = 0.17). However, there was some evidence of non-proportionality for patients in the lesion length categories 150–199 mm and > 300 mm after adjusting for all other factors in the model.

**Table 3:** Univariate Cox model results

Characteristic^a^	Hazard ratio (95% CI)	*p* value
Male	0.699 (0.567, 0.861)	< 0.001*
Age		
65–74	0.791 (0.622, 1.004)	0.049*
75–84	0.734 (0.565, 0.954)	
> 85	0.558 (0.293, 1.062)	
Diabetes	1.141 (0.932, 1.396)	0.202
Hypertension	0.884 (0.676, 1.157)	0.370
Hypercholesterolemia	1.089 (0.880, 1.347)	0.434
Renal disease	1.340 (1.070, 1.677)	0.011*
Smoking status		
Past	0.726 (0.570, 0.925)	0.024*
Current	0.918 (0.704, 1.196)	
Rutherford		
CLI	1.845 (1.439, 2.365)	< 0.001*
Lesion length		
50–99 mm	1.607 (1.122, 2.300)	< 0.001*
100–149 mm	2.367 (1.649, 3.398)	
150–199 mm	2.416 (1.551, 3.763)	
200–249 mm	3.501 (2.392, 5.126)	
250–299 mm	3.897 (2.576, 5.897)	
> 300 mm	5.130 (3.381, 7.784)	
RVD		
≥ 5 mm	0.808 (0.649, 1.007)	0.057
Popliteal involvement	1.451 (1.041, 2.021)	0.028*
Total occlusion	1.882 (1.536, 2.305)	< 0.001*
Calcification		
Mild/moderate	0.953 (0.749, 1.213)	0.752
Severe	1.054 (0.772, 1.439)	
Prior interventions	2.124 (1.720, 2.624)	< 0.001*
Number of runoff vessels		
≥ 2	0.866 (0.695, 1.078)	0.198

* CLI* critical limb ischemia; *RVD* reference vessel diameter

*Statistically significant, *p* < 0.05

^a^Reference levels are—Sex: Female; Age: < 65; Diabetes: No; Hypertension: No; Hypercholesterolemia: No; Renal Disease: No; Smoking status: Never; Rutherford: Claudicant; Lesion length: < 50 mm; RVD: < 5 mm; Popliteal involvement: No; Total occlusion: No; Calcification: None; Prior interventions: No; Number of runoff vessels: ≤ 1

**Table 4:** Multivariate Cox model results

Characteristic^a^	Hazard ratio (95% CI)	*p *value
Male	0.760 (0.600, 0.961)	0.022*
Age		
65–74	0.734 (0.573, 0.941)	0.002*
75–84	0.637 (0.481, 0.844)	
> 85	0.398 (0.206, 0.771)	
Diabetes	1.033 (0.835, 1.277)	0.766
Hypertension	0.927 (0.700, 1.228)	0.596
Hypercholesterolemia	1.126 (0.901, 1.407)	0.296
Renal disease	1.072 (0.838, 1.372)	0.578
Smoking status		
Past	0.825 (0.629, 1.083)	0.187
Current	1.020 (0.753, 1.383)	
Rutherford		
CLI	1.429 (1.091, 1.872)	0.010*
Lesion length		
50–99 mm	1.443 (1.003, 2.074)	< 0.001*
100–149 mm	2.066 (1.425, 2.994)	
150–199 mm	2.205 (1.398, 3.478)	
200–249 mm	2.847 (1.886, 4.299)	
250–299 mm	2.899 (1.848, 4.547)	
> 300 mm	3.454 (2.159, 5.526)	
RVD		
≥ 5 mm	0.727 (0.578, 0.914)	0.006*
Popliteal involvement	1.042 (0.737, 1.473)	0.815
Total occlusion	1.406 (1.118, 1.769)	0.004*
Calcification		
Mild/moderate	0.994 (0.777, 1.271)	0.845
Severe	1.078 (0.781, 1.488)	
Prior interventions	1.815 (1.443, 2.282)	< 0.001*
Number of runoff vessels		
≥ 2	0.958 (0.756, 1.213)	0.719

*CLI* critical limb ischemia;* RVD* reference vessel diameter

* Statistically significant, *p* < 0.05

^a^Reference levels are—Sex: Female; Age: < 65; Diabetes: No; Hypertension: No; Hypercholesterolemia: No; Renal Disease: No; Smoking status: Never; Rutherford: Claudicant; Lesion length: < 50 mm; RVD: < 5 mm; Popliteal involvement: No; Total occlusion: No; Calcification: None; Prior interventions: No; Number of runoff vessels: ≤ 1

**Fig. 2:**
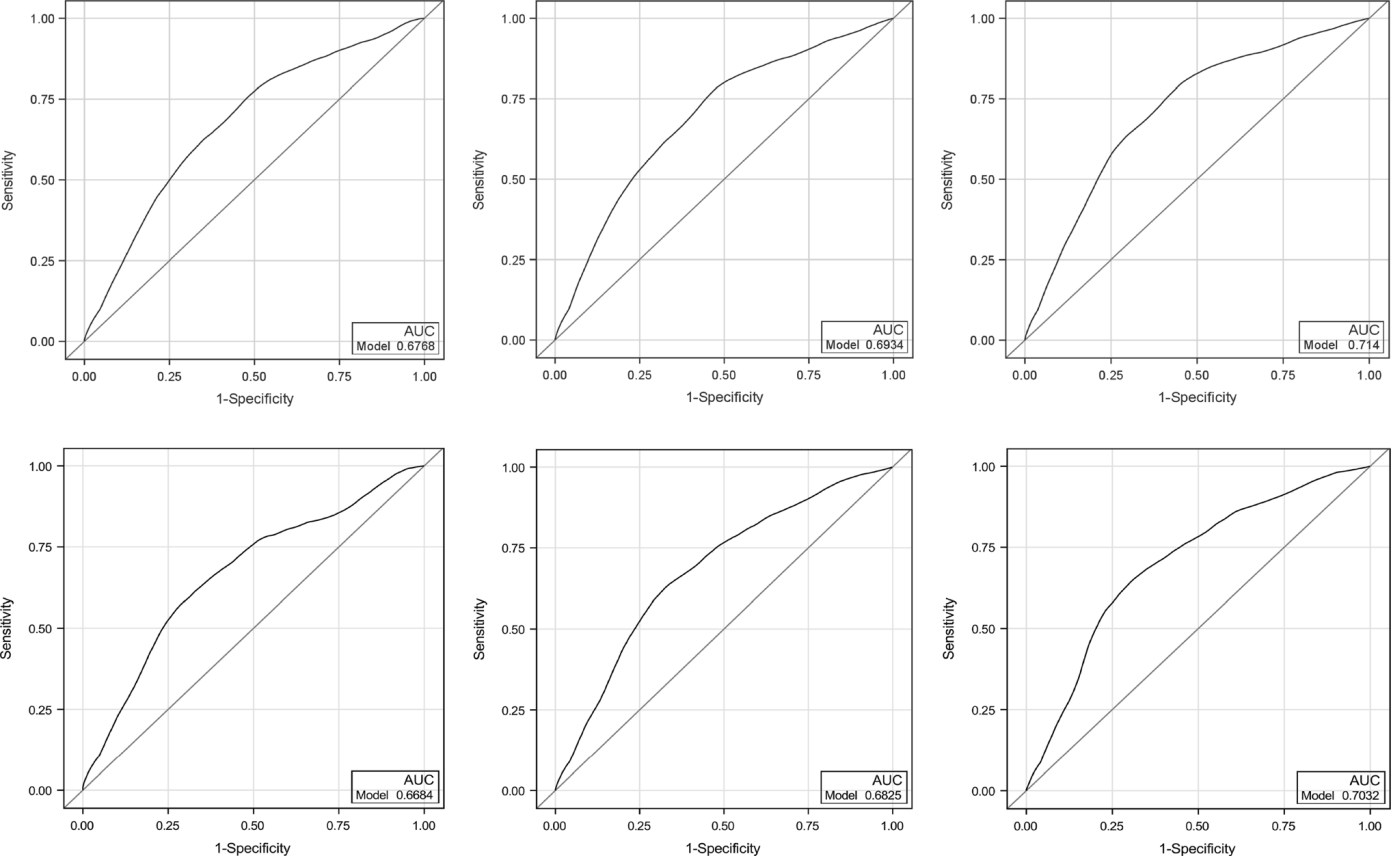
Receiver operating curves for the complete data set and for the training data set. ROCs for the complete data set (top panel) and for the training data set (bottom panel) at 1, 3, and 5 years show very similar performance. ROC, receiver operating curves

**Fig. 3:**
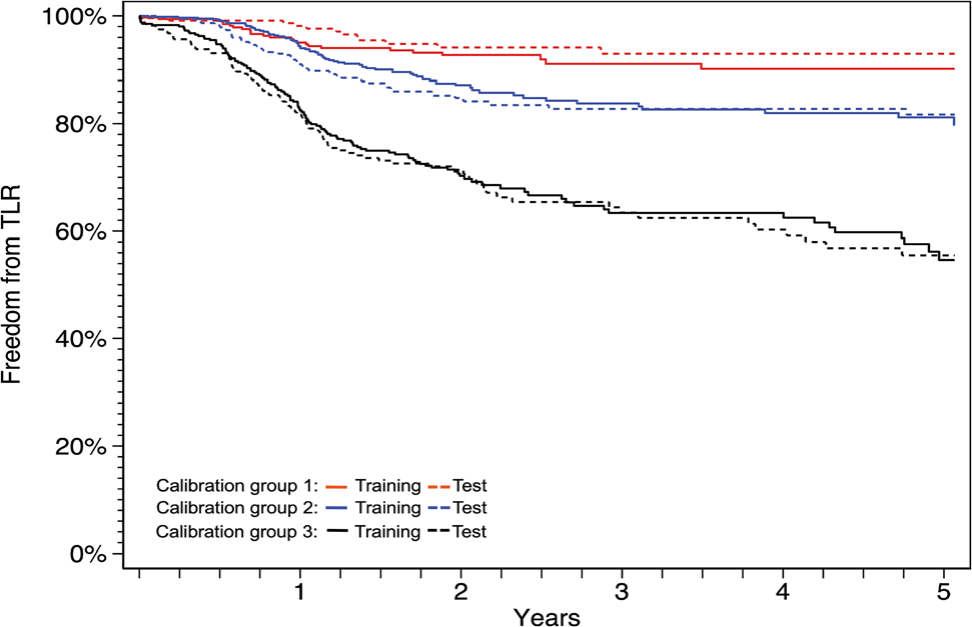
Calibration curves for training data set versus test data set. Calibration groups were defined by cuts at the 27th and 73rd percentiles of the distribution of the linear predictor [13]. The solid lines represent the training data set for each of the three calibration groups; the dashed lines represent the test data sets for each of the three calibration groups

ROCs for the complete data set and for the training data set at 1, 3, and 5 years show very similar performance (Fig. 2), with area under the curve (AUC) approximately 0.70 for all timepoints. The c-statistic and associated 95% CI based on the test data set was 0.70 (0.66, 0.74), suggesting adequate discrimination of the model [14]. Figure 3 shows good calibration for all three groups. Based on the validation results and the finding that the training model parameters were similar in direction and magnitude to the parameters from the complete model, prediction results are reported from the model using the complete dataset.

**Fig. 4:**
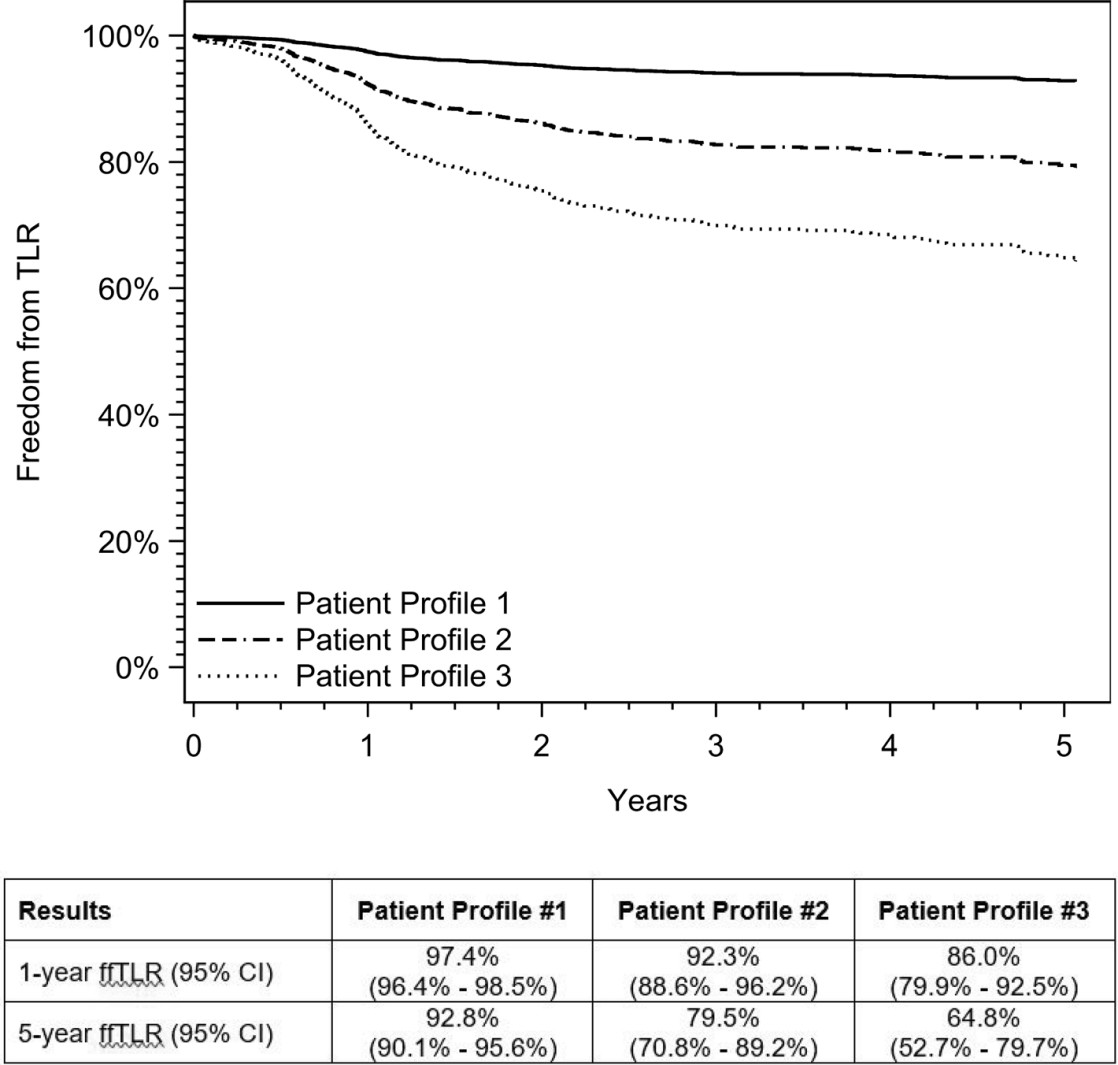
Predicted freedom from TLR for example patient profiles. Curves for freedom from TLR correspond to the patient characteristics outlined in Table 5. Patient profile #1 is shown in red. Patient profile #2 is shown in blue. Patient profile #3 is shown in yellow. The predicted rates of freedom from TLR for each patient profile are included in the table. TLR, target lesion revascularization

Examples of three representative patient profiles are shown in Table 5. Commonly observed patient and lesion characteristics were selected in the model resulting in a range of ffTLR through 5 years for the three representative patients. The predicted freedom from TLR curves corresponding to these patient profiles is shown in Fig. 4. Through 1 year, example patient profile 1 has a predicted freedom from TLR of 97.4% (95% CI: 96.4%–98.5%) and a predicted freedom from TLR of 92.8% (95% CI: 90.1%–95.6%) through 5 years. Example patient profile 2 has predicted freedom from TLR of 92.3% (95% CI: 88.6%–96.2%) and 79.5% (95% CI: 70.8%–89.2%) through 1 year and 5 years, respectively. The predicted freedom from TLR for example patient profile 3 is 86.0% (95% CI: 79.9%–92.5%) and 64.8% (95% CI: 52.7%–79.7%) through 1 year and 5 years, respectively.

**Table 5:** Example of patient profiles

Factor	Patient profile #1	Patient profile #2	Patient profile #3
Sex	Male	Female	Male
Age	65–74	65–74	75–84
Diabetes	Yes	Yes	No
Hypertension	Yes	Yes	Yes
Hypercholesterolemia	Yes	Yes	No
Renal disease	No	No	Yes
Smoking status	Past smoker	Past smoker	Past smoker
Rutherford classification	Claudicant	Claudicant	Claudicant
Lesion length	< 50 mm	100–149 mm	200–249 mm
RVD	≥ 5 mm	≥ 5 mm	≥ 5 mm
Popliteal involvement	No	No	No
Occlusion	No	No	Yes
Calcification severity	Mild/moderate	Severe	Mild/moderate
Prior interventions	No	No	Yes
Number of runoff vessels	2 +	0 or 1	2 +

*RVD* reference vessel diameter

## Discussion

PAD affects the lower extremities and is associated with functional decline, reduced quality of life, increased depression, and increased cardiovascular morbidity and mortality [15]. Repeat treatment for PAD is also costly, inconvenient, and associated with procedural risks. As population management becomes more important, the balance between upfront procedural cost and the cost of caring for the patient in an “extended episode of care” becomes more important. The results of the data analysis identified that certain traditional risk factors commonly thought to influence reintervention rates did not have an impact, but other clinical and anatomic variables did affect the risk of TLR after DES placement.

The present study with an aggregated data analysis of 2227 patients with femoropopliteal PAD treated with the DES is the largest to date that examines the outcomes of a drug-based endovascular device according to the presence of patient and lesion risk factors. An analysis of the impact of these risk factors on ffTLR through 5 years of follow-up allowed creation of a prediction model. This prediction model provides a risk profile for individual patients that should markedly improve risk assessment for TLR over standard clinical variables. This model can help facilitate discussions between physicians and patients with PAD regarding expected outcomes with this DES therapy. More generally, it may contribute to improving the cost-effectiveness of care by allowing vascular specialists to optimize patient selection for DES treatment.

As anticipated, there is some variability in the frequency of individual factors between studies. In some instances, this is most likely due to differences in study enrollment criteria. For example, the Japan PMS and SAS studies enrolled longer lesions, which is reflective of the studies not excluding lesions based on length. In contrast, the other three studies limited enrollment to a maximum lesion length of 140 mm. Smoking status, hypercholesterolemia (relatively low in the China study), and prior interventions were more frequent in the Japan PMS and SAS studies. Patients with ISR were enrolled in the Japan PMS and SAS studies in contrast to the other three studies where patients with prior stent placement were excluded. Inclusion of longer lesions and ISR strengthens the validity of the model predictions across a diverse patient population that is representative of patients commonly treated in clinical practice.

In both the univariate and multivariate analyses, the factors that were identified as significant were expected predictors of TLR including gender, age, CLI, lesion length, RVD, total occlusion, and prior interventions which includes ISR. Recent publication of 3-year results of DCB found similar risk of TLR including lesion length, RVD ≤ 4.5 mm, ISR, bilateral disease, CLI, and hyperlipidemia [16]. Although popliteal involvement and smoking were significant in the univariate analysis, these factors were no longer significant when adjusting for other risk factors. A strong correlation between popliteal involvement and RVD is expected, and when including both in the model, RVD remained significant. This finding is consistent with observations from DCB [17]. The frequency of a RVD > 5 mm was more common in the Japan PMS and SAS studies. It is possible that the differences reflect regional variations in the use of intravascular ultrasound (IVUS) rather than conventional angiography to determine vessel size. Reliance on IVUS for definitive determination of RVD is a common practice and deemed a more accurate representation of true vessel size than the traditional depiction of only the lumen provided by conventional arteriography [18]. A study from Japan found a significantly higher rate of freedom from reintervention through 5 years when IVUS was used compared to non-use of IVUS [19].

There were several factors that are thought to be significant predictors of TLR; however, in this model, these factors were not significant predictors for TLR of the DES. The presence of renal disease and the number of standard tibial runoff arteries, traditionally believed to negatively impact ffTLR, did not show a significant impact in the presence of other factors included in the model. These unanticipated results are in agreement with what has been previously reported for the DES [20, 21]. While diabetes mellitus is commonly considered a risk factor for TLR following standard PTA and BMS, in this analysis diabetes did not have a significant impact, a finding previously reported in analyses of DES [22].

Patient age had a significant impact on TLR but in an unanticipated way. In this analysis, there was a decreasing risk of TLR as age increased. Although this may be counterintuitive initially, there are some possible explanations for this effect. Patients in the older age group may not be as ambulatory as younger patients. If patients are sedentary, they may not experience claudication or pain associated with exercise. In addition, older individuals in general have more medical comorbidities and may not be considered good candidates for reintervention. Although the finding was for restenosis and not TLR, analysis of the bare metal Supera stent showed an increased risk of restenosis through 3 years for patients < 75 years old compared to patients > 75 years old [23]. This finding is consistent with the results for age from the prediction model in which older patients had a decreased risk of TLR.

This prediction model has been adapted into an online application for clinical use by physicians (https://cooksfa.z13.web.core.windows.net). Using a patient’s unique risk profile, which is based on the patient’s personal clinical and lesion characteristics, the calculator predicts the risk of TLR over time. The calculator can help guide patient selection for DES placement by estimating individual risk of TLR through 5 years following SFA treatment with DES. In this study, three representative patient profiles with commonly observed patient and lesion characteristics demonstrated the functionality of this approach. In addition, this may stimulate similar modeling efforts focused on other endovascular devices with data from clinical trial experience that provides long-term follow-up. Such studies may help the field define an optimal treatment algorithm for custom management of an individual with symptomatic SFA PAD and lead to evidence-based guidance focused on maximizing the benefits and cost effectiveness of intervention.

The study and the prediction model are associated with inherent limitations. Not all of the studies have 5-year follow-up (three out of five studies with 5-year results). Incomplete data collection of all factors in this analysis was minimal, resulting in approximately 6% of patient cases being omitted from the analysis. The omitted cases had a higher proportion of patients with an RVD ≥ 5 mm. These patients would be expected to have a higher ffTLR rate than those patients with smaller RVDs; therefore, the model may potentially underestimate ffTLR. Other patient characteristics were not eligible for inclusion because data for these factors were not collected in each of the five studies, including the precise type of diabetes, pulmonary dysfunction, and prior myocardial infarction. In addition, core lab analysis provides the best available data for evaluation of most anatomic factors; however, a core lab was not utilized in all studies. Accounting for all other factors in the model, the proportionality assumption was not met for two of the seven lesion length groups. Both lesion groups have considerable numbers of patients from the Japan PMS study (> 45% of the 150–199 mm lesion length group and almost all of the ≥ 300 mm lesion length group). The authors speculate that this, along with the uniqueness of the ≥ 300 mm lesion length group, could contribute to the assumption not being met. Violation of this assumption suggests that a more complex relationship than what is currently presented may be relevant for this small subset of patients. Overall, because the study outcomes and prediction model are derived from a post hoc patient-level pooled analysis from five prospective clinical trials, the present results should be considered hypothesis generating.

In summary, this study evaluates a drug-based endovascular device used for management of SFA PAD and the factors that predict ffTLR for the DES. The study uses an extensive dataset to develop the first prediction model which estimates the influence of patient and lesion characteristics on ffTLR through 5 years. Based on a unique patient profile, the model provides expected patient outcomes following treatment with the Zilver PTX DES. The results from this prediction model may assist physicians to define treatment algorithms for patients as population management grows in focus.

## Electronic supplementary material

Below is the link to the electronic supplementary material.

Supplementary file1 (DOCX 14 kb)
