# Supplementary material for: Prediction Model for Freedom from TLR from a Multi-study Analysis of Long-Term Results with the Zilver PTX Drug-Eluting Peripheral Stent
Source: Cardiovasc Intervent Radiol. 2020 Oct 6;44(2):196–206. doi: 10.1007/s00270-020-02648-6 (PMC7806559; doi:10.1007/s00270-020-02648-6)
Supplement: Supplementary file 1 — Supplementary file1 (DOCX 14 kb) [file 270_2020_2648_MOESM1_ESM.docx]

**Supplementary Table 1. Comparison of complete cases versus omitted cases**

| **Characteristic** | | **Complete** | **Omitted** | **p-value** |
| --- | --- | --- | --- | --- |
| Sex | Male | 71.1%  (1583/2227) | 68.7%  (101/147) | 0.539 |
|  | Female | 28.9%  (644/2227) | 31.3%  (46/147) |  |
| Age | <65 | 30.0%  (668/2227) | 23.4%  (34/145) | 0.212 |
|  | 65-74 | 37.7%  (839/2227) | 36.6%  (53/145) |  |
|  | 75-84 | 28.2%  (627/2227) | 34.5%  (50/145) |  |
|  | >85 | 4.2%  (93/2227) | 5.5%  (8/145) |  |
| Diabetes | | 48.9%  (1090/2227) | 44.2%  (65/147) | 0.267 |
| Hypertension | | 83.8%  (1867/2227) | 83.7%  (123/147) | 0.959 |
| Hypercholesterolemia | | 61.2%  (1362/2227) | 57.1%  (84/147) | 0.334 |
| Renal disease^b^ | | 22.8%  (508/2227) | 25.9%  (38/147) | 0.396 |
| Smoking status | Never | 25.8%  (574/2227) | 27.3%  (33/121) | 0.867 |
|  | Past | 46.3%  (1030/2227) | 43.8%  (53/121) |  |
|  | Current | 28.0%  (623/2227) | 28.9%  (35/121) |  |
| Rutherford | Claudicant | 85.4%  (1902/2227) | 81.8%  (54/66) | 0.417 |
|  | CLI | 14.6%  (325/2227) | 18.2%  (12/66) |  |
| Lesion length | <50 mm | 25.8%  (574/2227) | 23.8%  (31/130) | 0.121 |
|  | 50-99 mm | 27.3%  (607/2227) | 17.7%  (23/130) |  |
|  | 100-149 mm | 17.9%  (399/2227) | 19.2%  (25/130) |  |
|  | 150-199 mm | 7.6%  (169/2227) | 10.8%  (14/130) |  |
|  | 200-249 mm | 9.9%  (220/2227) | 12.3%  (16/130) |  |
|  | 250-299 mm | 6.7%  (149/2227) | 7.7%  (10/130) |  |
|  | >300 mm | 4.9%  (109/2227) | 8.5%  (11/130) |  |
| RVD | <5 mm | 28.3%  (630/2227) | 15.8%  (22/139) | 0.001* |
|  | ≥5 mm | 71.7%  (1597/2227) | 84.2%  (117/139) |  |
| Popliteal involvement | | 7.8%  (173/2227) | 6.1%  (9/147) | 0.468 |
| Total occlusion | | 42.1%  (937/2227) | 43.4%  (63/145) | 0.745 |
| Calcification | None | 23.3%  (519/2227) | 28.8%  (42/146) | 0.293 |
|  | Mild/moderate | 59.0%  (1314/2227) | 56.2%  (82/146) |  |
|  | Severe | 17.7%  (394/2227) | 15.1%  (22/146) |  |
| Prior interventions | | 21.0%  (467/2227) | 18.4%  (27/147) | 0.452 |
| Number of runoff vessels | 0-1 | 28.6%  (638/2227) | 31.1%  (41/132) | 0.552 |
|  | >2 | 71.4%  (1589/2227) | 68.9%  (91/132) |  |

CLI, critical limb ischemia; RVD, reference vessel diameter

^a^ Best available data was used since a core lab was not utilized in all studies

^b^ The status for renal disease was collected as yes/no for all studies except the China study where it was collected as “chronic renal failure” (n=0), “dialysis” (n=0) or “other renal disease” (n=10). The sum of these three measured were considered for renal disease status for the China study.

* Statistically significant, p<0.05
